# Stem-Centered Drought Tolerance in *Mikania micrantha* During the Dry Season

**DOI:** 10.3390/ijms26199722

**Published:** 2025-10-06

**Authors:** Minling Cai, Minghao Chen, Junjie Zhang, Changlian Peng

**Affiliations:** 1 College of Life Science, Huizhou University, Huizhou 516007, China; cmlscnu@163.com (M.C.); cmh950108@163.com (M.C.); 2 Guangzhou Key Laboratory of Subtropical Biodiversity and Biomonitoring, Guangdong Provincial Key Laboratory of Biotechnology for Plant Development, College of Life Sciences, South China Normal University, Guangzhou 510631, China; takozhang0444@163.com

**Keywords:** *M. micrantha*, stem, dry season, osmotic adjustment substance

## Abstract

*Mikania micrantha*, commonly known as mile-a-minute weed, is listed among the world’s top 10 worst weeds. Although native to humid regions of South America, it has recently been found to colonize arid habitats as well. Despite pronounced seasonal hydroclimatic variations in South China and increasing drought due to global climate change, the mechanisms underlying *M. micrantha*’s drought tolerance remain poorly understood. In this study, we compared the photosynthetic responses of *M. micrantha* leaves and stems between the dry (June) and wet (December) seasons through field experiments. We measured changes in phenotype, photosynthetic characteristics, and the content of antioxidant and osmotic adjustment substances, using the co-occurring native vine *Paederia scandens* as a control. The results revealed that during the dry season, *M. micrantha* leaves exhibited wilting, along with significant reductions in relative water content (RWC), chlorophyll (Chl), soluble sugar (SS), and soluble protein (SP). In contrast, the stems of *M. micrantha* maintained relatively stable phenotypes and chlorophyll levels compared to those of *P. scandens*. Notably, *M. micrantha* stems exhibited significant increases in vessel wall thickness, vessel density, total phenol content, and the activities of peroxidase (POD) and ascorbate peroxidase (APX). Furthermore, compared to *P. scandens*, *M. micrantha* stems displayed a greater increase in cortex proportion, flavonoid content, and soluble protein content. Expression analysis of *bZIP* transcription factors further revealed drought-responsive upregulation of specific genes (*bZIP60*, *ZIP42-1*), suggesting their potential involvement in drought response. These results indicate that although the leaves of *M. micrantha* are susceptible to prolonged drought, the stems exhibit considerable resilience, which may be attributed to a combination of traits including structural modifications in stem anatomy, enhanced antioxidant capacity, and osmotic adjustment. These insights suggest that stem-specific adaptations are key to its drought tolerance, providing a theoretical foundation for understanding the habitat distribution of *M. micrantha* and informing effective management strategies.

## 1. Introduction

With the ongoing development of globalization and the expansion of human activities, increasing CO_2_ emissions and atmospheric concentrations have intensified the Earth’s greenhouse effect, posing an inevitable environmental challenge [1]. Studies predict that global average temperatures will rise by at least 1.5 °C to 3 °C over the next century [2]. Climate change is expected to alter precipitation patterns, leading to disparities in water resources across different regions and the expansion of arid areas, particularly in Southwest China. Drought, resulting from reduced precipitation, is one of the most common natural hazards affecting this region [3,4,5]. At the same time, biological invasions have intensified with the changes in the global environment [6,7,8]. *Mikania micrantha*, a highly invasive exotic weed originating from tropical Central and South America [9], has invaded numerous forests in South China, causing significant economic and environmental consequences [10]. Although it is typically found in wet forest areas and freshwater swamps in its native habitat [11], *M. micrantha* has shown remarkable adaptability to dry soils and shady environments [9], enabling it to thrive and spread even in increasingly dry conditions caused by global climate change. Previous studies have demonstrated that drought conditions reduce the content of photosynthetic pigments (chlorophyll a [Chla], chlorophyll b [Chlb], and total chlorophyll [Chl]) and gas exchange (daily mean of photosynthetic rate [P_day_], transpiration rates [T_r_], stomatal conductance [G_s_]) in *M. micrantha* leaves [12]. Additionally, Zhang et al. reported that prolonged drought triggers metabolic adjustments in *M. micrantha*, including increased proline and malondialdehyde (MDA) content, as well as enhanced activity of superoxide dismutase (SOD), catalase (CAT), and glutathione (GSH) [13]. These findings suggest that while drought suppresses photosynthetic efficiency and exacerbates oxidative stress in *M. micrantha*, the species exhibits compensatory mechanisms to sustain metabolic balance. However, the possible mechanism of *M. micrantha* successfully invading South China with distinct dry and wet seasons, especially the dry-season habitat, has not yet been elucidated. *Paederia scandens* (Lour.) Merr., a common native congener, has frequently been used as a control species in studies on *M. micrantha* [14,15,16]. To elucidate the invasive strategies of *M. micrantha* during dry seasons, this study investigates seasonal variations in leaf and stem morphology, vascular bundle structure, antioxidant capacity, and pigment and osmotic substance content in both *M. micrantha* and *P. scandens* in South China. Recent advances in drought stress management, particularly in staple crops, highlight the importance of physiological, biochemical, and molecular adaptations to water scarcity. As reviewed by Khan et al. [17], plants employ a range of strategies including osmotic adjustment, antioxidant defense, phytohormone regulation, and morphological modifications to mitigate drought-induced damage. These mechanisms are crucial for maintaining cellular integrity and metabolic function under water-limited conditions. Understanding such adaptive responses provides a valuable framework for interpreting how invasive species like *M. micrantha* cope with seasonal drought, potentially informing future management strategies in vulnerable ecosystems.

## 2. Results

### 2.1. Change in Phenotypes and Water Content During Dry and Wet Seasons

Soil moisture content showed significant seasonal variation (*p* < 0.001, Table 1) with wet season levels measuring approximately 10.6-fold higher than dry season values. Phenotypic observations (Figure S1) exhibited vigorous growth during the wet season, with *M. micrantha* demonstrating a greater advantage over *P. scandens*. In contrast, both species showed severe leaf chlorosis, wilting, and abscission in the dry season, particularly for *M. micrantha*. Notably, *M. micrantha* stems maintained structural integrity without visible wilting. The results also showed that unlike *P. scandens*, the leaf relative water content (RWC) of *M. micrantha* significantly decreased by approximately 0.86 times during the dry season compared to the wet season. There were no significant changes in the stems of either plant (Table 1 and Table S2, ANOVA: species, treatment and species × treatment *p* < 0.001).

### 2.2. Changes in Stem Structural Characteristics During Dry and Wet Seasons

Cross-sectional analysis revealed distinct stem morphologies in two species. *M. micrantha* exhibited an irregular hexagonal stem shape, whereas *P. scandens* maintained an oval cross-section in both wet and dry seasons (Figure S2). During the dry season, both species showed a significant expansion in cortex proportion (Table S2, ANOVA: species, treatment *p* < 0.001), with *M. micrantha* increasing by 25.03% and *P. scandens* by 20.19% compared to the wet season. Notably, *M. micrantha* displayed pronounced xylem modifications, with vessel wall thickness (ANOVA: species *p* < 0.001, treatment *p* = 0.022, species × treatment *p* = 0.019) and vessel density (ANOVA: species *p* < 0.001, treatment *p* = 0.03, species × treatment *p* = 0.043) increasing approximately 1.3-fold and 1.9-fold, respectively. In contrast, *P. scandens* exhibited no significant seasonal variation in these traits. The central cylinder (pith) proportion of *M. micrantha* decreased significantly during the dry season, yet it remained 1.3 times greater than that of *P. scandens*. Xylem analysis indicated no significant interspecific or seasonal differences in vessel diameter or the ratio of vessel number to vascular bundle number (Table 2).

### 2.3. Changes in Pigment Content During Dry and Wet Seasons

**Figure 1:**
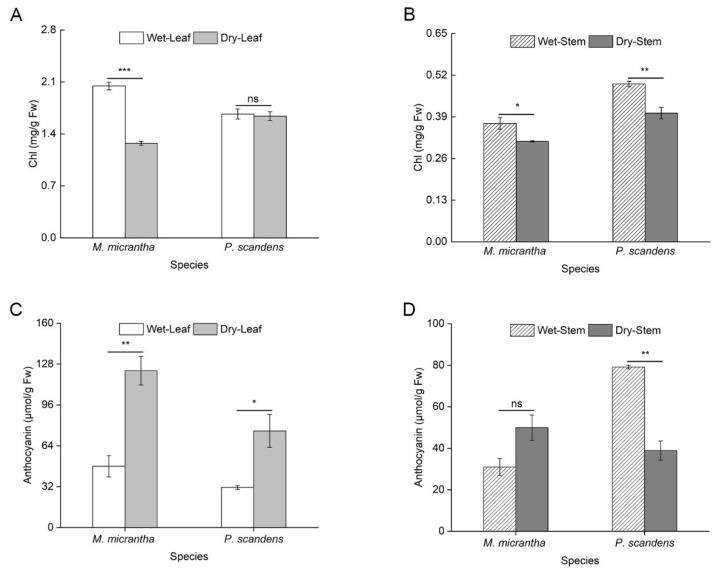
Changes in pigment content in leaves and stems of *M. micrantha* and *P. scandens* in wet and dry seasons. (**A**,**B**) represent Chl content; (**C**,**D**) represent anthocyanin content. Data are presented as mean ± standard error (n = 5). Asterisks indicate significant differences in the plant between two seasons, * *p* < 0.05, ** *p* < 0.01, *** *p* < 0.001, ns means not significantly.

Leaf chlorophyll (Chl) content in *M. micrantha* decreased significantly by 37.75% during the dry season, whereas *P. scandens* exhibited no notable change (Figure 1A, ANOVA: treatment *p* < 0.001, species × treatment *p* < 0.001). In stems, *M. micrantha* showed a moderate Chl reduction (15.20%), slightly less pronounced than that of *P. scandens* (18.47%; Figure 1B). Conversely, leaf anthocyanin content increased markedly in both species during the dry season. *M. micrantha* displayed a more substantial rise (155.77%) compared to *P. scandens* (140.83%; Figure 1C). However, while *P. scandens* displayed a significant decline in stem anthocyanin content, *M. micrantha* showed no detectable seasonal variation in this regard (Figure 1D, ANOVA: species, position, treatment *p* < 0.001, species × position × treatment *p* > 0.05).

### 2.4. Changes in Antioxidant Capacity During Dry and Wet Seasons

**Figure 2:**
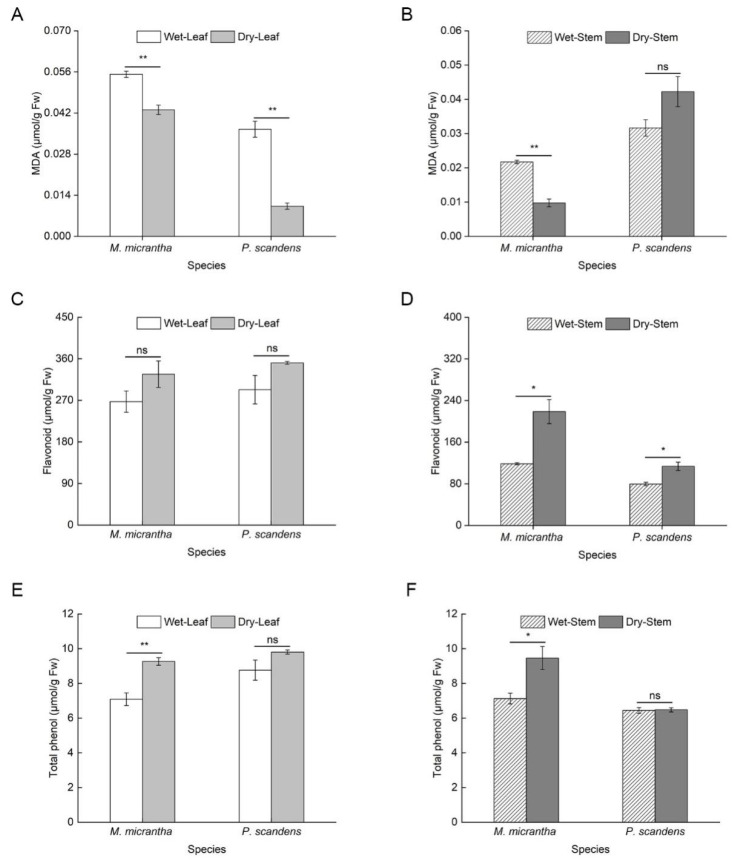
Changes in MDA content (**A**,**B**), flavonoid content (**C**,**D**) and total phenolic content (**E**,**F**) in leaves and stems of *M. micrantha* and *P. scandens* in wet and dry seasons. Data are presented as mean ± standard error (n = 5). Asterisks indicate significant differences in the plant between two seasons, * *p* < 0.05, ** *p* < 0.01, ns means not significantly.

**Figure 3:**
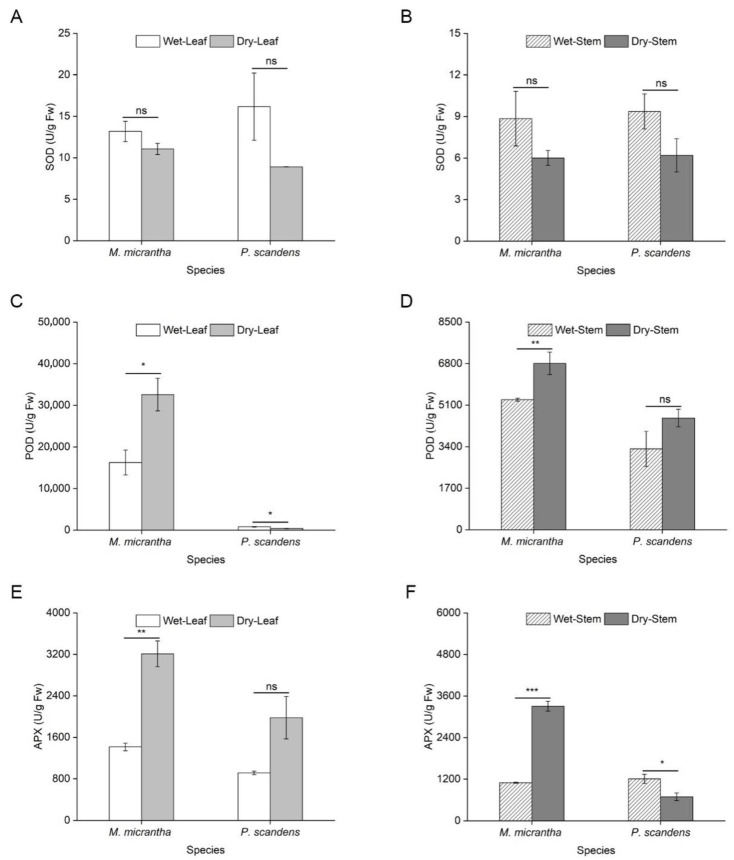
Changes in activity of antioxidant enzyme in leaves and stems of *M. micrantha* and *P. scandens* in wet and dry seasons. (**A**,**B**) represent SOD; (**C**,**D**) represent POD; (**E**,**F**) represent APX. Data are presented as mean ± standard error (n = 5). Asterisks indicate significant differences in the plant between two seasons, * *p* < 0.05, ** *p* < 0.01, *** *p* < 0.001, ns means not significantly.

During the dry season, Malondialdehyde (MDA) content decreased significantly in both species, with an 81% reduction in *M. micrantha* and a 30% decline in *P. scandens* leaves (Figure 2A). A similar trend was observed in stems, where *M. micrantha* showed decreased MDA levels, while *P. scandens* exhibited a slight increase (Figure 2B, ANOVA: species, treatment *p* < 0.001, species × treatment *p* < 0.001). Supporting these findings, electrolyte leakage measurements showed a parallel and significant decrease in both species and tissues during the dry season (Table S1). Specifically, in leaves, electrolyte leakage decreased from 30.63% to 22.41% in *M. micrantha* and from 28.13% to 16.13% in *P. scandens*. Similarly, in stems, electrolyte leakage declined from 24.96% to 17.03% in *M. micrantha* and from 25.55% to 16.31% in *P. scandens* (ANOVA: species, treatment, and position *p* < 0.001). No significant seasonal differences in leaf flavonoid content were detected in either species (Figure 2C). However, stem flavonoid content increased markedly during the dry season, with *M. micrantha* showing a 143.76% rise compared to 42.62% in *P. scandens* (Figure 2D, ANOVA: species = 0.033, position and treatment *p* < 0.001). Similarly, total phenolic content in *M. micrantha* leaves and stems was significantly higher in the dry season, whereas *P. scandens* showed no seasonal variation (Figure 2E,F, ANOVA: position, treatment, and species × treatment *p* < 0.01). SOD activity remained stable across seasons in both species (Figure 3A,B, ANOVA: position and species × treatment *p* > 0.05). In contrast, POD (ANOVA: species, position, and treatment *p* < 0.001) and APX (ANOVA: species and position *p* < 0.01) activities increased significantly in *M. micrantha* leaves and stems during the dry season, while *P. scandens* displayed opposing trends (Figure 3C,D).

### 2.5. Changes in Osmotic Adjustment Substances During Dry and Wet Seasons

**Figure 4:**
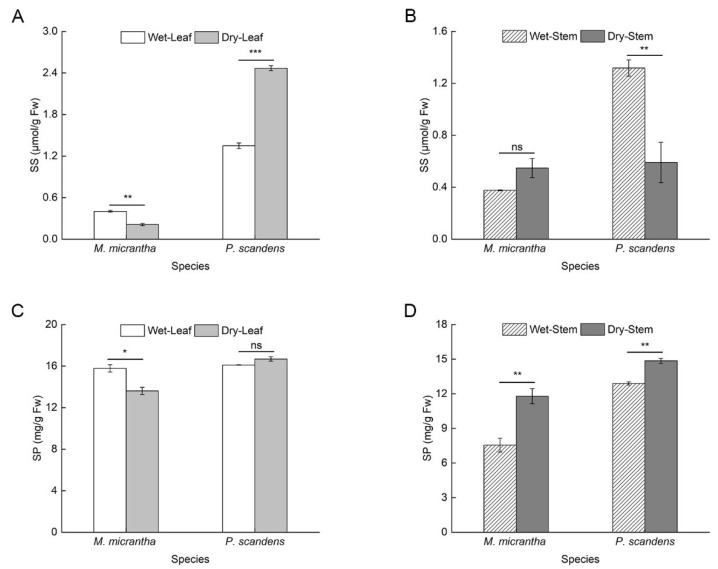
Changes in the content of osmotic adjustment substances in leaves and stems of *M. micrantha* and *P. scandens* in wet and dry seasons. (**A**,**B**) represent soluble sugar (SS); (**C**,**D**) represent soluble protein (SP). Data are presented as mean ± standard error (n = 5). Asterisks indicate significant differences in the plant between two seasons, * *p* < 0.05, ** *p* < 0.01, *** *p* < 0.001, ns means not significantly.

*M. micrantha* exhibited a significant reduction (25%) in soluble sugar (SS) content of leaves during the dry season compared to the wet season. Conversely, *P. scandens* leaves exhibited a significant increase in SS content during the dry season (Figure 4A). In stems, *M. micrantha* showed no significant seasonal variation in SS content, whereas *P. scandens* displayed a notable decline during the dry season (Figure 4B, ANOVA: species, treatment and species × treatment *p* < 0.001). During the dry season, the soluble protein (SP) content in the leaves of *M. micrantha* decreased significantly by about 84%, while *P. scandens* leaves showed no significant change (Figure 4C). Additionally, the increase in SP content in *M. micrantha* stems (56.11%) was greater than that observed in *P. scandens* stems (15.24%) (Figure 4D, ANOVA: species, treatment, and position *p* < 0.001, species × position × treatment *p* < 0.001).

### 2.6. Expression Patterns of bZIP Transcription Factors in Response to Drought and Their Tissue Specificity

**Figure 5:**
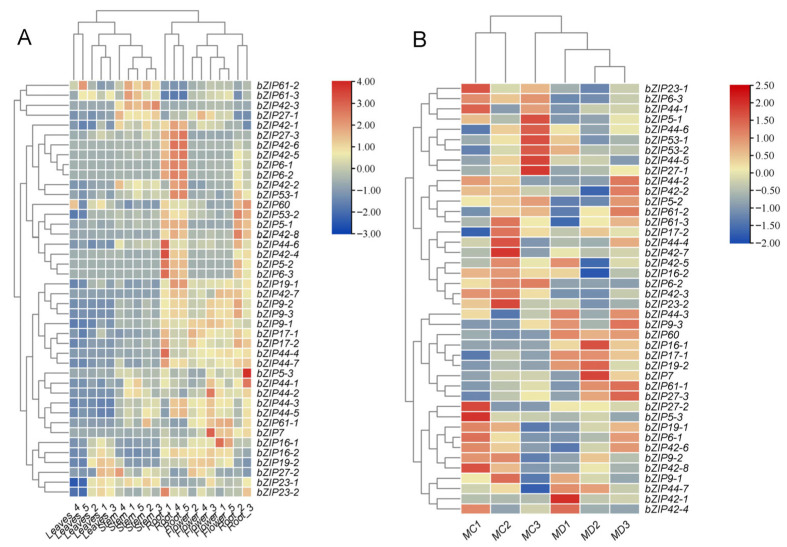
Expression analysis of *bZIP* gene family in *M. micrantha*. (**A**) represents the expression patterns of *bZIP* gene family in different tissues (flower, leaves, stem and root) of *M. micrantha*; (**B**) represents *bZIP* gene expression in stem of *M. micrantha* under drought treatments (MC1–MC3: control group; MD1–MD3: drought group).

To further elucidate the molecular mechanisms underlying the physiological changes under drought stress, we analyzed the expression patterns of the *bZIP* transcription factor family in *M. micrantha*. Heatmap analysis revealed that multiple *bZIP* genes exhibited significant differential expression under drought conditions, with distinct tissue-specific expression profiles. Tissue-specific expression profiling (Figure 5A) showed that the *bZIP* family genes exhibited diverse expression patterns, with most genes (such as *bZIP17*, *bZIP44*, and *bZIP9*) showing high expression in flowers and roots. A small number of genes, such as *bZIP60*, were highly expressed in leaves and roots, while genes including *bZIP23-1*, *bZIP42-1/2/3*, and *bZIP61-2/3* were predominantly highly expressed in stems. Furthermore, analysis of *bZIP* gene expression patterns under drought stress induction revealed diverse response characteristics across subfamilies (Figure 5B). Some genes were up-regulated under drought conditions (e.g., *bZIP60*, *bZIP42-1*, *bZIP61-1*), while others showed higher expression levels under well-watered conditions (e.g., *bZIP23-1*, *bZIP53-1*).

## 3. Discussion

As global climate change intensifies, biological invasions have become increasingly severe. In subtropical Southwest China, the climate is characterized by distinct wet (April–September) and dry (October–March) seasons [18]. Such pronounced seasonality in precipitation strongly influences soil moisture dynamics and plant physiological performance [19]. In our field experiment, we collected two wild vines, *M. micrantha* and a native plant, *P. scandens,* during dry and wet seasons. Soil moisture content in their habitats varied significantly, with higher levels during the wet season (Table 1), consistent with findings by Zhang et al. [20]. This moisture fluctuation directly influenced plant relative water content. During the dry season, *M. micrantha* leaves exhibited wilting and senescence due to reduced RWC (Figure S1; Table 1), suggesting weaker drought resistance compared to *P. scandens*. This aligns with studies showing that invasive species may exhibit high phenotypic plasticity but often display tissue-specific variations in stress tolerance [18,20]. Interestingly, while leaves suffered, *M. micrantha* stems remained turgid and healthy (Figure S1), indicating that stem adaptations may be crucial for its dry-season survival. This tissue-specific stress resistance strategy is likely orchestrated at the transcriptional level. Basic leucine zipper motif (*bZIP*) transcription factors play an important regulatory role in plant drought stress responses. Our study found that multiple *bZIP* transcription factors (such as *bZIP23-1*, *bZIP42-1/2/3*, etc.) were specifically highly expressed in stems (Figure 5A), suggesting that they may be key regulators in shaping stem identity and mediating its drought response [21].

### 3.1. Stem Structural Optimization Enhances Water Retention

The stem is a vital organ for water transport from roots to aerial tissues [22,23]. In *M. micrantha*, the cortex and medulla proportions increased during the dry season (Figure S2; Table 2). Li et al. demonstrated that the larger cortex and medulla areas enhance the water storage capacity of *Cerasus humilis* tissue, enabling plants to regulate water retention in arid environments and improve overall resistance, particularly in the stems [24]. The anatomical studies on *Tagetes erecta* also support these findings [25], indicating that the presence of parenchyma tissue with a higher proportion in the stem of *M. micrantha* enhances its water storage capacity. Research has shown that tannin acid, acting as a potent reducing agent in the medulla, could improve the ability of plants to scavenge oxygen free radicals generated under water stress, thereby minimizing oxidative damage caused by dehydration [26]. The xylem vessels are fundamental structures responsible for water transport in plants [27]. Research on the stem structure of *Carya illinoensis* across different climate types showed that varieties exhibiting strong drought tolerance generally have higher vessel density and thicker vessel walls in the cross-section of their stems, especially in areas with lower precipitation [28]. Similarly, the stem of *M. micrantha* exhibited increased vessel wall thickness and vessel density, indicating enhanced water transmission capacity during the dry season (Table 2). This adaptation mechanism potentially reduces vessel diameter by increasing vessel wall thickness, thereby preventing vessel cavitation and embolism [29,30]. Notably, these anatomical modifications are likely driven by specific *bZIP* transcription factors. For example, *bZIP42-1* and *bZIP61-1* were significantly upregulated under drought stress (Figure 5B) and may activate genes involved in cell wall synthesis and modification. Therefore, optimizing the stem structure and improving water transport capacity represent important strategies employed by *M. micrantha* to cope with drought stress.

### 3.2. Balance and Distribution of Photosynthetic Pigments

Chlorophyll degradation under drought is well-documented [31], often due to ROS-induced chloroplast damage [32]. In our observations, while the stem Chl content remained stable in both species (Figure 1B), the leaves of *M. micrantha* exhibited a significant loss of Chl, unlike those of *P. scandens* (Figure 1A). This reduction in Chl content typically leads to a decline in photosynthetic capacity under drought conditions [33]. The notable chlorophyll degradation in the leaves of *M. micrantha* during the dry season suggests poorer drought resistance, which may negatively affect its photosynthetic capacity. Conversely, anthocyanins increased in *M. micrantha* stems and both species’ leaves during the dry season (Figure 1). Cirillo et al. found that anthocyanins play an important role in enhancing plant drought resistance [34]. These findings indicate that anthocyanins could shield light energy and reduce ROS accumulation caused by excessive light [35,36]. Additionally, anthocyanins function as antioxidants, directly minimizing ROS accumulation in plants [37]. *BZIP* transcription factors are widely involved in regulating plant secondary metabolism. Studies have shown that overexpression of *NnbZIP36* in *Arabidopsis* promoted anthocyanin accumulation by upregulating key anthocyanin biosynthesis genes (including *4CL*, *CHI*, *CHS*, *F3H*, *F3’H*, *DFR*, *ANS*, and *UF3GT*) [38]. Consistent with this, our study observed a significant drought-induced increase in anthocyanin content in both the leaves and stems of *M. micrantha* (Figure 1C,D). This increase may potentially be regulated upstream by certain drought-responsive *bZIP* members, such as the drought-induced *MmbZIP60* (Figure 5B). These transcription factors likely enhance the plant’s photoprotection and antioxidant capacity by activating key enzyme genes in the anthocyanin biosynthesis pathway.

### 3.3. Accumulation of Antioxidant Substances

Drought stress typically induces substantial accumulation of reactive oxygen species (ROS) in plants [38], leading to membrane lipid peroxidation [36,39]. While numerous studies have reported drought-induced increases in MDA content in species such as *Nicotiana tabacum* [40] and *Sphagneticola species* [20], our results revealed an unexpected but consistent decrease in both MDA levels and electrolyte leakage during the dry season across most tissues (Figure 2A,B, Table S2). This concerted decline in two key indicators of membrane damage strongly suggests that efficient ROS detoxification, potentially mediated by enhanced production of antioxidant proteins [20]. Plants employ a dual antioxidant defense system to mitigate ROS under drought stress, consisting of non-enzymatic antioxidants (flavonoid and total phenol) and antioxidant enzymes (SOD, APX, POD, etc.) [41,42,43]. Our study showed higher levels of flavonoids and total phenols in *M. micrantha* compared to *P. scandens* (Figure 2C–F), consistent with previous findings on antioxidant content changes under drought conditions [44]. For instance, recent studies have highlighted that flavonoids and phenolic compounds act as crucial scavengers of ROS, thereby enhancing oxidative stress tolerance and supporting plant growth under water deficit [45]. These results suggested that the increasing antioxidant content enhances plant resistance by reducing ROS accumulation due to drought stress. Additionally, antioxidant enzymes also play a crucial role in removing excessive ROS to maintain intracellular balance [46,47]. Interestingly, while SOD activity remained unchanged between species and seasons (Figure 3A,B), *M. micrantha* exhibited marked increases in POD and APX activities in both leaves and stems (Figure 3C–F). This enzymatic profile suggests that POD and APX serve as the primary ROS-scavenging systems in *M. micrantha*, efficiently converting H_2_O_2_ to H_2_O and maintaining redox homeostasis. Similar drought-responsive upregulation of antioxidant enzymes has been documented in *Catharanthus roseus* [48], *Sphagneticola trilobata* [20], and *Vitis vinifera* [49]. Despite the above evidence, the observed reduction in membrane damage markers remains physiologically atypical. We acknowledge that other factors—such as tissue-specific ROS dynamics, compensatory metabolic adaptations, or yet-unidentified non-oxidative pathways—may also contribute to the observed phenomena. Future research involving direct measurement of specific ROS like H_2_O_2_ could provide more precise insights into the oxidative stress status under these conditions. Study have shown that *OsbZIP62* improves drought and oxidative tolerance in rice [50]. Complementing these antioxidant mechanisms, osmotic adjustment through soluble sugar and soluble protein accumulation represents another critical drought adaptation strategy [51], consistent with recent findings across various plant species [52,53]. Notably, while *M. micrantha* leaves showed limited osmotic adjustment capacity, its stems demonstrated substantial increases in SS and SP content during drought, surpassing levels observed in *P. scandens* (Figure 4). This stem-specific response highlights a key physiological differentiation within *M. micrantha*, with stems exhibiting stronger osmotic adjustment ability compared to leaves during water-limited conditions.

## 4. Materials and Methods

### 4.1. Plant Growth and Collection

The subtropical climate in Southwest China exhibits distinct wet (April to September) and dry seasons (October to March) [18]. Two plants in this study including *M. micrantha* and *P. scandens* were grown in the campus of South China Normal University (23°10′ N, 113°21′ E). *P. scandens* was chosen as a control species because field surveys revealed that it not only occurs frequently and stably in communities invaded by *M. micrantha* but also shares similar reproductive strategies with *M. micrantha* [54]. The phenotypic characteristics of *M. micrantha* and *P. scandens* were observed during wet season (June 2021) and dry season (December 2021) using digital cameras (Sony, α6000, Sony Imaging Products & Solutions Inc., Tokyo, Japan). Stem segments from the first to the sixth nodes of both plants, which exhibited similar growth patterns, were collected to analyze relevant physiological indicators in this experiment. Additionally, soil samples from the plant habitat were collected to determine soil water content. The plant samples were rinsed with tap water to remove soil and surface debris, followed by a wash with distilled water. All the samples were frozen in liquid nitrogen and stored at −80 °C for further analysis.

### 4.2. Determination of Relative Water Content (RWC)

The relative water content (RWC) of plant tissues was determined using a modified protocol described by Ogbaga et al. [55]. A fully expanded leaf (1 piece) and stem segment (1 cm long) were weighed to obtain their respective fresh weight (FW). The samples were then immersed in 5 mL of distilled water within a centrifuge tube for 24 h to achieve full turgidity, after which their turgid weight (TW) was recorded. Finally, the samples were oven-dried at 75 °C until a constant dry weight (DW) was attained. RWC was calculated using the following formula:

RWC (%) = (FW − DW)/(TW − DW) × 100%.

Soil moisture content was measured following the oven-drying method [18]. Briefly, 10 g of fresh soil was weighed (SW1) and dried at 75 °C until a constant mass (SW2) was achieved. The soil moisture content was calculated as:

Soil moisture content (%) = (SW1 − SW2)/SW1 × 100%.

### 4.3. Observation of Stem Structure

Stem samples from both plant species were collected during dry and wet seasons and processed using standard paraffin methods [56]. A total of over twenty cross-sections (representing at least five biological replicates per species per season) were prepared and scanned using a high-resolution digital slide scanner (Hamamatsu S360, Hamamatsu Photonics K.K., Hamamatsu, Japan). Multiple anatomical parameters were quantified using ImageJ version 1.54p software. The proportion of cortex, medulla and duct density were calculated using the following formula: cortex proportion (%) = (cortex area/slice area) × 100%; medulla proportion (%) = (medulla area/section area) × 100%; catheter density (Individual/mm^2^) = (catheter number/slice area).

### 4.4. Determination of Chlorophyll and Anthocyanin Content

Fresh leaf and stem samples (0.05 g) were homogenized in 4 mL of 80% acetone and extracted overnight at 4 °C. Following centrifugation at 4 °C, 3000 rpm for 10 min, the absorbance of the supernatant was measured at 663 nm, 645 nm and 470 nm using 80% acetone as a blank. Chlorophyll a, b, and total chlorophyll concentrations were calculated according to Wellburn [57]. Anthocyanin content was determined following Cai et al. [36] with modifications. Samples (0.05 g) were homogenized in 2 mL of methanol:HCl (99:1, *v*/*v*) solution and extracted at 4°C in the dark for 24 h. The reaction mixture, containing 0.5 mL deionized water, an equal volume of supernatant, and chloroform, was analyzed at 532 nm using a UV-Vis 2450 spectrophotometer (Shimadzu, Kyoto, Japan). The chlorophyll and anthocyanin content were expressed as (μg/g FW) and (μmol/g FW), respectively.

### 4.5. Determination of Malondialdehyde (MDA) and Soluble Sugar (SS) Content

MDA content was determined using the thiobarbituric acid (TBA) method [58]. Tissue samples (0.05 g) were homogenized in 2 mL of 0.1% (*w*/*v*) trichloroacetic acid (TCA) solution. After centrifuged at 4 °C, 1000 rpm for 10 min, 1 mL of the supernatant was mixed with an equal volume of 0.67% (*w*/*v*) TBA solution. The mixture was incubated at 95 °C for 30 min in a water bath, and absorbance was measured at 532 nm, 600 nm, and 645 nm using a spectrophotometer. The MDA and SS content were expressed as (μmol/g FW).

### 4.6. Determination of Activities of Enzymatic Antioxidants and Soluble Protein Content

Fresh tissue samples (0.05 g) were homogenized in 1 mL of extraction buffer (consist of 50 mM Phosphate buffer [PBS], 0.1 M Ethylenediaminetetraacetic acid [EDTA], Triton-X-100 and polyvinylpyrrolidone [PVP], pH 7.8). The homogenate was centrifuged at 12,000× *g* for 10 min at 4 °C, and the supernatant was collected for enzymatic analyses. SOD activity was determined using a modified nitroblue tetrazolium (NBT) reduction method [59]. One unit of SOD activity is defined as the amount of enzyme required to inhibit nitro blue tetrazolium (NBT) photoreduction by 50%. A final volume of 3 mL of the mixture (consisted of 50 mM PBS, 20 μM riboflavin, 130 mM methionine, 0.1 μM EDTA, 750 mM NBT, and 100 μL of enzyme extract) was recorded at a wavelength of 560 nm. POD activity was assayed according to Du et al. [60] using a reaction mixture containing 50 mM PBS (pH 7.0), 30 mM H_2_O_2_, guaiacol, and 100 μL enzyme extract. The increase in absorbance at 470 nm was recorded for 3 min. APX activity was measured following Nakano and Asada [61] by monitoring the oxidation of ascorbate at 290 nm for 1 min at 10 s intervals. The antioxidant enzyme activities were calculated and expressed as (U/g FW). The soluble protein content was determined using the bicinchoninic acid (BCA) method. A diluted aliquot of the enzyme extract was mixed with a 2× Bradford dye reagent. The absorbance was measured at 595 nm using a spectrophotometer. A standard curve was prepared using a protein standard solution, and the soluble protein content was calculated and expressed as (mg/g FW).

### 4.7. Determination of Flavonoid and Total Phenol Content

Flavonoid and total phenol contents were estimated by the method of [62], Ainsworth and Gillespie with slight modifications, respectively [63]. 0.05 g sample were homogenized in 2 mL of 95% (*v*/*v*) methanol, the supernatant was obtained after centrifuged at 12,000 rpm for 10 min. Flavonoid content was determined following a modified method of Park et al. [61]. The reaction mixture was composed of 2 mL supernatant (diluted 8-fold), 0.2 mL of NaNO_2_ (5%, *v*/*w*), 0.3 mL of AlCl_3_ (10%, *v*/*w*) and 1 mL of 1 M NaOH. After thorough mixing, absorbance was measured at 510 nm against a methanol blank. Total phenolic content was quantified using the Folin–Ciocalteu method with modifications [62]. The absorbance of reaction mixture (equal volume of supernatant and Folin–Ciocalteu (10%, *w*/*v*) were mixed with 0.7 M Na_2_CO_3_) were measured at 765 nm using a Uv-Vis 2450 spectrophotometer. The flavonoid and total phenol content were expressed as (μmol/g FW).

### 4.8. Screening and Identification of bZIP Gene Family Members in M. micrantha

The genomic information, annotation files, and protein sequence data of *M*. *micrantha* were downloaded from the NCBI website (http://www.ncbi.nlm.nih.gov/ (accessed on 20 August 2025)). The protein sequences of the *Arabidopsis thaliana AtbZIP* gene family were retrieved from the TAIR database and used as query sequences for BLAST version 2.16.0 alignment against the *M*. *micrantha* database. The E-value was set to 10^−8^, with a similarity threshold of >60%, to preliminarily screen candidate genes. The presence of conserved domains (F00170, PF07716) was confirmed using the CD-search and SMART [64].

### 4.9. Expression Pattern Analysis of bZIP Gene Family Members in M. micrantha

According to the method of Chen et al. [64], transcriptome data from the database were used to analyze the expression of *M*. *micrantha* bZIP genes in different organs (roots, stems, leaves, and flowers) and under drought induction treatment. RNA-seq datasets (No. SRR8857621–SRR8857640) were obtained from the SRA database (https://www.ncbi.nlm.nih.gov/sra (accessed on 20 August 2025)) on the NCBI website. The raw data underwent quality control, including adapter removal and redundancy filtering, to generate clean data for subsequent alignment and quantitative analysis. TPM or FPKM values were used to measure gene expression levels. R Studio (version 4.4.2) was employed to generate heatmaps illustrating the expression of *MmbZIP* gene family members in different organs and under drought treatment.

### 4.10. Data Analysis

All data are presented as mean ± standard deviation (SD) from three to five biological replicates. Differences between dry and wet seasons of the same species (*M. micrantha* or *P. scandens*) were detected by Studentʼs t-test using IBM SPSS^®^ Statistics (Version 19.0, IBM Corporation, New York, NY, USA) software. Data visualizations were carried out using OriginPro (Version 8.0, OriginLab Corporation, Northampton, MA, USA) and Adobe Photoshop CC (Version 2017, Adobe Systems, San Jose, CA, USA) software.

## 5. Conclusions

In summary, our findings reveal a striking dichotomy in *M. micrantha’*s drought response between its leaf and stem tissues. During the dry season, *M. micrantha* leaves exhibit a significantly reduced RWC, compromised phenotypic integrity, lower levels of chlorophyll, and decreased osmotic adjustment capabilities compared to the native *P. scandens*. However, this apparent vulnerability is balanced by the exceptional adaptive features of the stem of *M. micrantha*, which include enhanced anatomical structures for water storage and transport, superior antioxidant defense systems, and robust osmotic regulation capabilities. These stem-specific characteristics, potentially mediated by *bZIP* transcription factors—though future molecular evidence is needed—may underpin *M. micrantha*’s remarkable drought resilience despite its foliar limitations. We hypothesize that this differential tissue specialization could represent a key evolutionary strategy facilitating the species’ successful establishment and ongoing invasion in subtropical South China. The ability of the stem to maintain physiological function during seasonal drought could provide *M. micrantha* with a competitive advantage over native vegetation, particularly in regions experiencing increasing aridity due to climate change. While our anatomical findings are suggestive of improved hydraulics, and the chlorophyll reduction hints at lower photosynthetic capacity, future studies incorporating direct measurements of stem hydraulic conductivity, cavitation vulnerability, and leaf gas exchange are needed to definitively establish these mechanistic links.

## Figures and Tables

**Table 1:** The RWC of leaves and stems of *M. micrantha* and *P. scandens* in dry and wet season and soil moisture content at the sampling site.

Plant Organ	*M. micrantha*		*P. scandens*		Soil Moisture Content (%)	
	Wet	Dry	Wet	Dry	Wet	Dry
Leaf-RWC (%)	88.68 ± 1.15	79.69 ± 0.19 **	92.14 ± 0.25	87.71 ± 1.63 ns	26.60 ± 0.96	2.52 ± 0.04 ***
Stem-RWC (%)	83.99 ± 0.45	81.31 ± 1.67 ns	94.75 ± 1.00	83.84 ± 4.17 ns		

**Note**: Data are presented as mean ± standard error (n = 5). Asterisks after the same row of data for each species represent significant differences between different seasons, ** means *p* < 0.01, *** means *p* = 0.00, ns means not significantly different, the same below.

**Table 2:** Statistical indexes of stem vascular cylinder of *M. micrantha* and *P. scandens* in dry and wet seasons.

Indicators	*M. micrantha*		*P. scandens*	
	Wet	Dry	Wet	Dry
Cortex proportion (%)	23.97 ± 1.01	29.97 ± 0.72 **	28.38 ± 1.27	35.56 ± 0.42 **
Medulla proportion (%)	47.8 ± 1.03	41.79 ± 1.34 *	37.35 ± 2.53	31.07 ± 0.52 ns
Catheter diameter (μm)	126.07 ± 13.69	91.79 ± 14.76 ns	109.97 ± 13.78	88.59 ± 3.77 ns
Catheter/vascular bundles (Individual/Individual)	3.05 ± 0.07	3.24 ± 0.23 ns	2.62 ± 0.06	2.77 ± 0.03 ns
vessel wall thickness (μm)	1.91 ± 0.13	2.4 ± 0.10 *	1.67 ± 0.04	1.67 ± 0.02 ns
Catheter density (Individual/mm^2^)	28.4 ± 2.36	53.89 ± 7.35 *	29.88 ± 6.19	30.97 ± 2.17 ns

**Note**: Data are presented as mean ± standard error (n = 5). Asterisks after the same row of data for each species represent significant differences between different seasons, * means *p* < 0.05, ** means *p* < 0.01, ns means not significantly different, the same below.

## Data Availability

All datasets for this study are included in the manuscript and/or the Supplementary Files.

## Supplementary Materials

The following supporting information can be downloaded at https://www.mdpi.com/article/10.3390/ijms26199722/s1.

## Abbreviations

The following abbreviations are used in this manuscript:

RWC	Relative Water Content
Chl	Chlorophyll
SS	Soluble Sugar
SP	Soluble Protein
POD	Peroxidase
APX	Ascorbate Peroxidase
SOD	Superoxide Dismutase
CAT	Catalase
GSH	Glutathione
FW	Fresh Weight
TW	Turgid Weight
DW	Dry Weight
MDA	Malondialdehyde
TBA	Thiobarbituric Acid
TCA	Trichloroacetic Acid
PBS	Phosphate Buffer
EDTA	Ethylenediaminetetraacetic Acid
PVP	Polyvinylpyrrolidone
NBT	Nitroblue Tetrazolium
SD	Mean ± Standard Deviation
