# Supplementary material for: Stem-Centered Drought Tolerance in Mikania micrantha During the Dry Season
Source: Int J Mol Sci. 2025 Oct 6;26(19):9722. doi: 10.3390/ijms26199722 (PMC12524506; doi:10.3390/ijms26199722)
Supplement: Supplementary file 1 [file ijms-26-09722-s001.zip › ijms-3862903-supplementary.pdf]

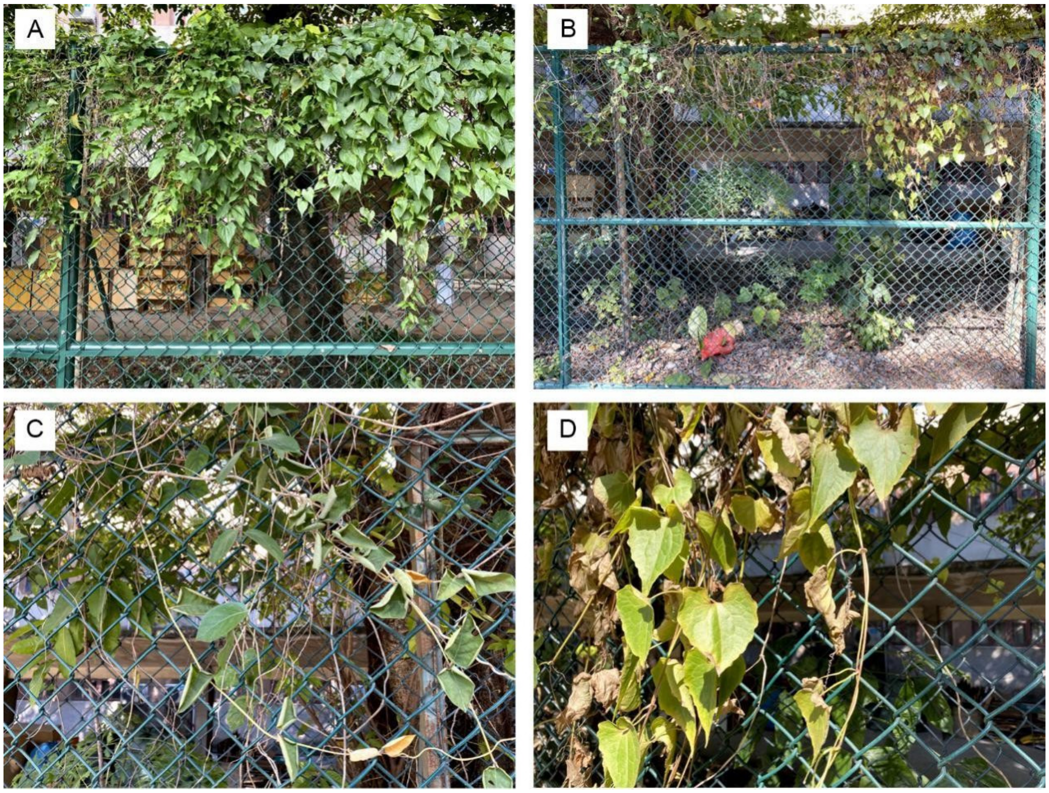

Figure S1. Phenotypic differences between *M. micrantha* and *P. scandens* in wet and dry seasons. A and B are the growth status of *M. micrantha* and *P. scandens* in wet and dry seasons, respectively; C and D are the phenotypes of leaves and stems of *P. scandens* and *M. micrantha* in dry season, respectively.

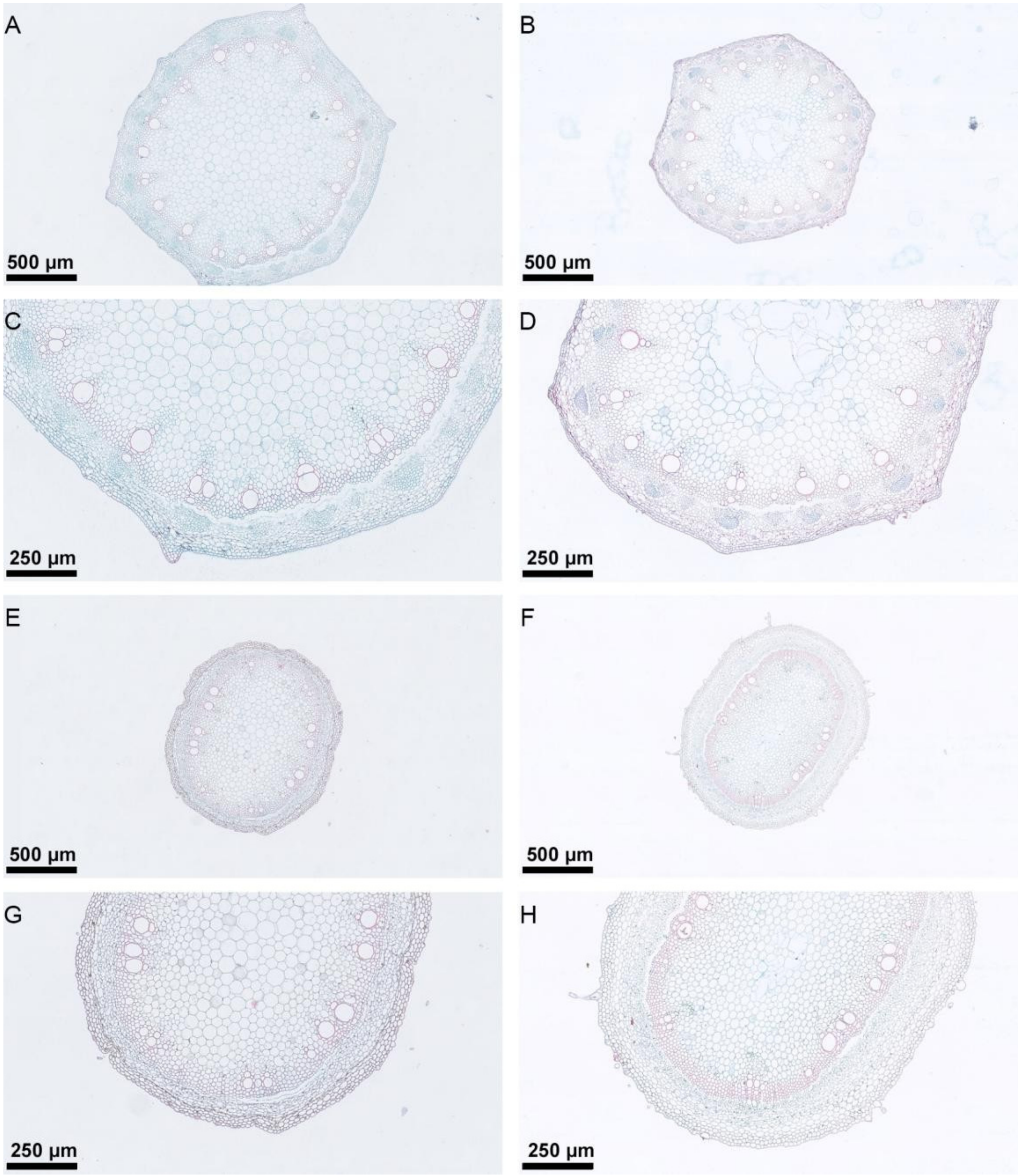

Figure S2. Paraffin sections of the stems of *M. micrantha* and *P. scandens* in dry and wet seasons. A and B are the stem sections of *M. micrantha* in wet season and dry season under 5 × objective lens, respectively; C and D are the stem sections of *M. micrantha* in wet season and dry season under 10 × objective lens, respectively; E and F are the stem sections of *P. scandens* in wet season and dry season under 5 × objective lens, respectively; G and H are the stem sections of *P. scandens* in wet season and dry season under 10 × objective lens, respectively. Black rectangles in A, B, E and F are 500 μm scale bars and black rectangles in C, D, G and H are 250 μm scale bars.

Table S1. Statistical indexes of electrolyte leakage of *M. micrantha* and *P. scandens* in dry and wet seasons.

Indicators	*M. micrantha*		*P. scandens*	
	**Wet**	**Dry**	**Wet**	**Dry**
Leaf（%）	30.63±0.78	22.41±1.72*	28.13±0.32	16.13±0.37***
Stem（%）	24.96±0.80	17.03±1.42***	25.55±0.63	16.31±1.09***

Note: Data are presented as mean ± standard error (n = 5). Asterisk after the same row of data for each species represent significant differences between different seasons, * means $p < 0.05$, ** means $p < 0.01$, *** means $p = 0.00$, ns means not significantly different, the same below.

Table S2 Variance analysis results of physiological indicators related to stems and leaves of *M. micrantha* and *P. scandens* in dry and rainy seasons.

Properties	df	F	P
Anthocyanin content (mmol g^{-1} FW)			
Species	1	8.912	0.009
Position	1	9.197	0.008
Treatment	1	16.841	0.001
Species*Position	1	52.16	0
Species*Treatment	1	43.349	0
Treatment*Position	1	42.728	0
Species*Treatment*Position	1	0.027	0.873
Flavonoid (μmol/g Fw)			
Species	1	5.411	0.033
Position	1	82.214	0
Treatment	1	20.925	0
Species*Position	1	14.14	0.002
Species*Treatment	1	3.835	0.068
Treatment*Position	1	1.524	0.235
Species*Treatment*Position	1	3.686	0.073
Total phenols (μmol/g Fw)			
Species	1	1.893	0.188
Position	1	26.382	0
Treatment	1	28.194	0
Species*Position	1	31.147	0
Species*Treatment	1	10.622	0.005
Treatment*Position	1	0.66	0.428
Species*Treatment*Position	1	1.232	0.284
Chlorophyll content (μg/g)			
Species	1	3.183	0.093
Position	1	2098.781	0
Treatment	1	74.071	0
Species*Position	1	4.183	0.058
Species*Treatment	1	41.339	0
Treatment*Position	1	35.203	0
Species*Treatment*Position	1	49.853	0
POD (U/g Fw)			
Species	1	194.614	0
Position	1	106.686	0
Treatment	1	35.713	0
Species*Position	1	13.822	0.002
Species*Treatment	1	74.583	0

Treatment*Position	1	11.392	0.004
Species*Treatment*Position	1	6.88	0.018
SOD (U/g Fw)			
Species	1	248.897	0
Position	1	0.092	0.766
Treatment	1	14.015	0.002
Species*Position	1	9.256	0.008
Species*Treatment	1	0	0.982
Treatment*Position	1	1.172	0.295
Species*Treatment*Position	1	0.446	0.514
APX (U/g Fw)			
Species	1	127.324	0
Position	1	11.044	0.004
Treatment	1	2.756	0.116
Species*Position	1	6.324	0.023
Species*Treatment	1	0.021	0.887
Treatment*Position	1	10.173	0.006
Species*Treatment*Position	1	2.307	0.148
MDA content (μmol/g Fw)			
Species	1	1617.229	0
Position	1	2.121	0.165
Treatment	1	40.66	0
Species*Position	1	40.604	0
Species*Treatment	1	227.788	0
Treatment*Position	1	1.853	0.192
Species*Treatment*Position	1	35.18	0
Soluble protein content (mg/g Fw)			
Species	1	10434.626	0
Position	1	121.791	0
Treatment	1	199.318	0
Species*Position	1	18.562	0.001
Species*Treatment	1	21.929	0
Treatment*Position	1	0.2	0.661
Species*Treatment*Position	1	53.086	0
Soluble sugar content (μmol/g Fw)			
Species	1	1427.159	0
Position	1	474.526	0
Treatment	1	69.175	0
Species*Position	1	3.766	0.07
Species*Treatment	1	133.198	0
Treatment*Position	1	4.577	0.048
Species*Treatment*Position	1	59.547	0
Relative water content (%)			
Species	1	18962.47	0

Position	1	24.257	0
Treatment	1	0.745	0.401
Species*Position	1	28.871	0
Species*Treatment	1	0.129	0.725
Treatment*Position	1	0.534	0.476
Species*Treatment*Position	1	0.001	0.975
Electrical conductivity (%)			
Species	1	4090.149	0
Position	1	9.897	0.006
Treatment	1	22.537	0
Species*Position	1	174.32	0
Species*Treatment	1	9.365	0.007
Treatment*Position	1	3.212	0.092
Species*Treatment*Position	1	1.166	0.296
Proline content (μg/g)			
Species	1	349.833	0
Position	1	148.602	0
Treatment	1	28.595	0
Species*Position	1	167.815	0
Species*Treatment	1	41.273	0
Treatment*Position	1	120.715	0
Species*Treatment*Position	1	37.051	0
Cortex proportion (%)			
Species	1	30.062	0.001
Treatment	1	52.153	0
Species*Treatment	1	0.42	0.535
Medulla proportion (%)			
Species	1	46.96	0
Treatment	1	15.819	0.004
Species*Treatment	1	0.008	0.933
Catheter diameter（μm）			
Species	1	0.612	0.457
Treatment	1	5.084	0.054
Species*Treatment	1	0.273	0.615
Catheter/vascular bundles（Individual / Individual）			
Species	1	13.252	0.007
Treatment	1	1.998	0.195
Species*Treatment	1	0.038	0.851
vessel wall thickness（μm）			
Species	1	33.092	0
Treatment	1	7.968	0.022
Species*Treatment	1	8.514	0.019
Catheter density（Individual/mm^2）			
Species	1	4.483	0.067

Treatment	1	6.881	0.03
Species*Treatment	1	5.804	0.043
